# How many skin barriers haveth we: Percutaneous egression of ions?

**DOI:** 10.1111/srt.13119

**Published:** 2021-11-09

**Authors:** Chavy Chiang, Howard I. Maibach

**Affiliations:** ^1^ School of Medicine and Dentistry University of Rochester Rochester New York USA; ^2^ Department of Dermatology University of California San Francisco California USA

**Keywords:** electrolyte efflux, percutaneous egression, skin electrolytes, transepidermal ion flux

## Abstract

**Introduction:**

Skin provides critical barrier properties that enable terrestrial life. Myriad research has focused on the “water barrier” to transepidermal water loss (TEWL) despite there being a multitude of skin barrier properties. We asked what other barrier properties may have been overlooked and compiled data demonstrating the “electrolyte barrier” to be of potential clinical relevance.

**Methods:**

A literature search was conducted through PubMed, Embase, Google Scholar, and Web of Science databases for the following keywords: “transepidermal” or “epidermal” or “cutaneous” or “skin” or “percutaneous” and “ion” or “sodium” or “chloride” or “potassium” or “electrolyte” and “flux” or “egression.” Textbooks at the University of California, San Francisco were also hand reviewed. Experimental studies quantifying in vivo or ex vivo percutaneous egression of ions in response to human skin barrier perturbation were included.

**Results:**

Experimental damage to skin, mostly by tape‐stripping, frequently induced increased ion flux rates through the epidermis, in addition to increases in TEWL values. Interestingly, barrier perturbation did not always result in a concomitant rise in TEWL and transepidermal ion flux rates, such as in delipidization, indicating a distinction between the two barriers.

**Conclusion:**

Quantifying the percutaneous egression of ions in response to physical or chemical alterations may offer additional data that are not to be captured with TEWL studies exclusively. Continued efforts should be made to: (1) advance this technique as a method of assessing skin status and (2) enhance our understanding of other barriers and mechanisms.

## 1 INTRODUCTION

Mammalian skin maintains certain barrier properties that are necessary to sustain human life, including those which impede water loss outwardly through skin, resist percutaneous penetration of harmful substances inwardly, and maintain antimicrobial properties to inhibit skin colonization by pathogenic microorganisms. To date, the vast majority of skin barrier research has largely focused on external water loss through the skin, referred to as transepidermal water loss (TEWL), while newer research focusing on the complex relationship between the cutaneous microbiome, the skin, and the skin's immune cells, termed the “immune barrier,” has burgeoned. In light of the extensive work already conducted to examine water loss through the skin, and that is currently being conducted to examine the skin microbiome, we propose that other cutaneous barriers of clinical relevance warrant further thought and investigation. Specifically, the barrier to the percutaneous egression, or the inside‐to‐outside movement of various substances through the skin,^1^ of ions deserves further exploration given their physiological importance within the context of bodily functioning. Whereas percutaneous penetration of ions in human skin is low,^2^ the percutaneous egression of ions can reach levels of clinical significance. Indeed, severe consequences secondary to electrolyte loss through the skin can occur in diseases that denude the cutaneous barrier, including *Staphylococcal* scalded skin syndrome, pemphigus vulgaris, and toxic epidermal necrolysis. Herein is a summary of the literature examining the percutaneous egression of ions as a potential marker of barrier integrity. However, another overlooked barrier to consider is that which influences cutaneous respiration, or CO_2_ and O_2_ gas exchange occurring through skin.^3^

## 2 MATERIALS AND METHODS

A literature search was conducted within PubMed, Embase, Google Scholar, and Web of Science databases. Keywords included “transepidermal” or “epidermal” or “cutaneous” or “skin” or “percutaneous” and “ion” or “sodium” or “chloride” or “potassium” or “electrolyte” and “flux” or “egression.” Textbooks at the University of California, San Francisco were hand reviewed. Only experimental articles quantifying the flux of ions across the epidermis in vivo or ex vivo on human skin/human skin samples were included. Note that the term “transepidermal ion flux” will be used interchangeably with “percutaneous egression of ions” to convey the same meaning.

## 3 RESULTS

Anjo and Maibach^4^ utilized a combination chloride electrode to quantitatively measure the outward rate of transepidermal chloride ion flux in vivo, with the chloride ion source likely originating from epidermal interstitial fluid. A plexiglass block with a cavity was attached to the various anatomical sites and received the electrode. The cavity was filled with sodium nitrate solution, and chloride transport measurements were made on the volar forearm, palm, dorsal hand, back, calf, and dorsal forearm of undisturbed skin. TEWL was measured by using ultrahigh purity nitrogen to direct a stream of gas through a sampling cup covering skin into a MEECO electrolytic moisture analyzer, where a phosphorus pentoxide film was used to absorb the water vapor before it was then quantified electrolytically. Additional chloride transport and TEWL measurements were taken after skin was disrupted by topical agents known to alter the stratum corneum, including dimethylsulfoxide (DMSO), acetone, 10% sodium dodecyl sulfate (sodium lauryl sulfate), ethyl acetate, and distilled water control, as well as cellophane tape‐stripping until the surface glistened. Measurements were also made on an individual with extensive anhidrotic burn scars and compared to a control to determine the contribution of eccrine sweat duct activity to TEWL and chloride transport.

Of the topical agents tested, DMSO was the only compound to significantly enhance chloride transport rates through the epidermis. Notably, Anjo and Maibach^4^ reported that enhanced chloride flux was detectable prior to visible erythema, and with no apparent change in TEWL, indicating the sensitivity of chloride transport rates over TEWL for the detection of this skin barrier disruption. Cellophane tape‐stripping, known to increase TEWL, was also found to significantly enhance chloride transport rates. The chloride transport rate in anhidrotic skin was within normal range, suggesting no significant contribution from eccrine gland activity in resting conditions free from obvious sweating. Based on these findings, there is a clear relationship observed between barrier function and the percutaneous egression of chloride. Citing the aforementioned sensitivity, Anjo and Maibach^4^ concluded chloride transport rates may prove to be a useful technique in determining stratum corneum integrity and presumably an independent barrier.

Grice et al.^5^ examined the effects of cellophane tape‐stripping on sodium and chloride concentrations in epidermal transudate using ion‐specific electrodes and compared this to the current benchmark for assessing skin barrier function, TEWL. TEWL was measured by passing nitrogen gas through a capsule covering 10 mm^2^ of skin and electrolytically measured (MEECO) for humidity. Other parameters measured were pH (H+ ion concentration) and galvanic skin resistance. Following sweat inhibition with a 4% poline methosulfate topical, in vivo tape‐stripping of human volar forearm commenced in 14 subjects. Measurements of the aforementioned parameters were taken every two to four strips and persisted until the cellophane tape no longer adhered to the skin. Parameters were also reassessed throughout a recovery period of several days post‐stripping.

Tape‐stripping increased TEWL values, from 1.2 mg/cm^2^/h to 60.8 mg/cm^2^/h, and pH values throughout the tape‐stripping period, from 4.6 to 6.6. Sodium and chloride ion concentrations in the epidermal transudate also increased, but did so sharply passed the halfway point of tape‐stripping, reaching extracellular fluid concentrations, from 1.7 to 126 mEq/L and 2.8 to 102 mEq/L, respectively. The point in which electrolyte concentrations sharply rose was also paralleled by a dramatic TEWL increase, suggesting simultaneous damage to both the water barrier and electrolyte barrier via tape‐stripping. Galvanic skin resistance decreased as expected, given the increasing concentration of electrolytes on the skin surface. All parameters returned to normal during the recovery period, achieving near‐normal values at 3–4 days post‐stripping. Grice et al.^5^ concluded this technique to be a quick and easy means of assessing water and electrolyte barrier function.

Interestingly, Grice et al.^6^ examined TEWL values (electrolytically, as above) and its sodium and potassium content in psoriatic skin compared to normal skin. Following eccrine sweat inhibition, the volar forearm of 15 normal subjects and six psoriatic subjects was thoroughly cleansed and dried. Dry, pre‐weighed filter papers covered by slightly larger polythene wrap with waterproof, adhesive edges were then placed on the forearm for 6 h in normal subjects and only 3 h in psoriatic subjects due to the increased TEWL observed in psoriasis. The filter paper was then re‐weighed and analyzed via flame photometry to determine sodium and potassium content.

TEWL values in psoriatic skin was higher, with a range of 2.0–5.6 mg/cm^2^/h, than in normal skin, of which the mean value measured 0.3 ± 0.1 mg/cm^2^/h. In contrast, all potassium concentrations and sodium concentrations in five of six psoriatic subjects were found to be within the range of normal skin ion concentrations. One psoriatic patient had an elevated sodium concentration within the collected epidermal transudate. The overall findings suggest a disruption to the epidermal water barrier that is not seen with the electrolyte barrier in patients with psoriasis.

Lo et al.^7^ assessed skin barrier function by examining TEWL and the percutaneous egression of chloride and potassium ions in normal, tape‐stripped, and delipidized stratum corneum. A combination chloride electrode and potassium ion electrode were used to do so in vivo on the volar forearm of human subjects, as well as an evaporimeter for TEWL. Seven healthy subjects were acclimatized to laboratory settings for at least 30 min before baseline values were taken. This was followed by cellophane tape‐stripping until surface glistening was achieved. Ten milliliter of a 1:1 acetone‐ether mixture was applied for 30 min to an adjacent area of skin for delipidization purposes. Measurements were taken afterward, and ion concentrations plotted against time. Linear regression analysis was utilized to generate a slope representative of ion flux rates, and analysis of variance between the three groups was performed for statistical comparison.

Delipidized skin only showed a significant difference in TEWL values, which was significantly increased when compared with normal skin. Tape‐stripped skin, however, showed a significant increase in all values with enhanced chloride flux, potassium flux, TEWL, and pH when compared to normal skin and suggests that delipidization solely disrupted the water barrier with no significant effect on the electrolyte barrier, which is in accordance with the findings of Grice et al.,^6^ whereas tape‐stripping incurred damage to both the stratum corneum water barrier and electrolyte barrier. Additionally, it is possible that ruptured epithelial cells and intracellular leakage secondary to tape‐stripping contributed to rising electrolyte levels. Importantly, the results of delipidization illustrate how an impaired water barrier does not necessarily equate to an impaired electrolyte barrier.

Jungman et al.^8^ examined the dermal to epidermal percutaneous egression of calcium ions in normal and tape‐stripped skin ex vivo with an ion‐specific electrode. TEWL was also measured using an evaporimeter. Human skin samples obtained from abdominal plastic surgery were placed onto Franz diffusion cells, with the epidermis facing the donor compartment and the dermis facing the receptor compartment. The epidermis‐donor compartment contained distilled water while the dermis‐receptor compartment was tested under changing conditions: either containing 100 mM CaCl_2_ solution or distilled water. A calcium electrode was placed into the epidermis‐donor compartment every half hour for 6 h to measure epidermal release of calcium ions. The system was then reversed via inversion of the skin samples, with the epidermis now facing the receptor compartment while the dermis faced the donor compartment. The epidermis‐receptor compartment contained distilled water, whereas the dermis‐donor compartment contained 100 mM CaCl_2_ solution. The calcium electrode was placed into the dermis‐donor compartment at equivalent time points to measure the dermal uptake of calcium ions by monitoring the loss of calcium ions in the dermis‐donor compartment.

Tape‐stripped skin showed significantly increased TEWL and released significantly more calcium ions into the epidermal‐donor compartment when compared to normal skin for both conditions, in which the dermal‐receptor compartment contained either distilled water or calcium solution. After 6 h, compared to normal skin, tape‐stripped skin released a three‐fold increase in total calcium ion concentration when paired with a distilled water source in the dermis‐receptor compartment, and a 33‐fold increase when paired with a calcium source in the dermis‐receptor compartment. Whereas no significant difference was observed between normal skins with water versus calcium source, tape‐stripped skin with a calcium source released a significant, 100‐fold increase in calcium ion concentration versus that with a water source. Following skin inversion, in which the dermis‐donor compartment contained a calcium source and the electrode while the epidermis‐receptor compartment contained a water source, dermal uptake of calcium ions between normal and tape‐stripped skin showed no significant difference in final concentrations after 6 h. Jungman et al.^8^ concluded that equivalent uptake of calcium ion concentrations occurred in both normal and tape‐stripped skin, but only tape‐stripped skin released increased amounts of calcium ions.

## 4 DISCUSSION

TEWL remains a principal focus of skin research, representing a popularly used parameter in examining skin barrier integrity. TEWL is extensively used across multiple research disciplines, including basic science, clinical studies, pharmacological studies, and cosmetic research.^9^ Although it remains a helpful and objective measure of barrier function, it does not represent the sole function of the skin barrier. Another property of the skin barrier that is similarly quantifiable is the maintenance of electrolyte contents within the body.

As demonstrated in the referenced studies, electrolyte concentration is easily detectable and may serve as a useful marker of skin barrier integrity. Table 1 summarizes each paper. Upon barrier disruption, most commonly via tape‐stripping but including delipidization in one study and the application of DMSO in another, the majority of studies show a detectable, concomitant increase in both TEWL, and the percutaneous egression of various ions captured by electrodes.^4^, ^5^, ^7^, ^8^ Additionally, Anjo and Maibach^4^ found that application of DMSO, an agent known to alter stratum corneum, enhanced chloride transport rates prior to visible erythema and without altering TEWL, implying superior sensitivity in the detection of barrier disruption by chloride transport rates when compared to the current gold standard of TEWL. These findings support the use of transepidermal ion flux as a potential marker of barrier integrity.

**TABLE 1:** Summary of studies evaluating the percutaneous egression of ions

Author	Ion(s) measured	Details
Anjo and Maibach^4^	Cl^–^	Cellophane tape‐stripping increased rate of transepidermal chloride flux and water loss. Treatment with DMSO application yielded a similar enhancement of chloride flux without affecting TEWL.
Grice et al.^5^	Na^+^ and Cl^–^	Tape‐stripping enhanced ionic efflux through skin and TEWL; both occurred at similar points in time, indicating simultaneous disruption to their respective barriers.
Grice et al.^6^	Na^+^ and K^+^	Affected areas of psoriatic skin were found to have similar rates of outward ion diffusion despite increases in TEWL values when compared to normal skin from healthy volunteers, suggesting a distinction between the epidermal water barrier and electrolyte barrier.
Lo et al.^7^	Cl^–^ and K^+^	Tape‐stripped skin had significantly increased rates of transepidermal ion flux and water loss compared to normal skin. Delipidized skin had significantly increased rates of water loss with no significant change in ion flux.
Jungman et al.^8^	Ca^2+^	Calcium release and uptake was assessed in tape‐stripped and normal skin samples placed in a Franz cell. Tape‐stripped skin released greater amounts of calcium ions through epidermis compared to normal skin, whereas both tape‐stripped skin and normal skin shared similar rates of dermal calcium uptake.

Abbreviations: DMSO, dimethylsulfoxide; TEWL, transepidermal water loss.

Interestingly, in addition to the DMSO skin challenge discussed above, two additional studies included experiments that did not demonstrate a simultaneous rise in both TEWL and transepidermal ion flux. Instead, a rise in TEWL accompanied by unaffected ion flux rates was observed. The first study examined TEWL as well as sodium and potassium ion flux in psoriatic skin compared to healthy skin.^6^ Although TEWL was significantly higher in affected plaques of psoriatic patients when compared to healthy skin from non‐affected individuals, transepidermal ion fluxes for potassium and sodium ions were normal in all and five of six psoriatic patients, respectively. This suggests that impairment of the water barrier does not always necessitate concomitant disruption to the electrolyte barrier. Lending credence to this idea, the acetone‐ether delipidization experiments performed by Lo et al.^7^ similarly reported a significant increase in TEWL with no significant changes in the percutaneous egression of potassium and chloride ions when compared to normal skin. This lack of simultaneous disruption in both studies might suggest that constituents of the water barrier are distinct from those of the electrolyte barrier.

Zhang and colleagues evaluating TEWL and skin conductance values from split‐thickness skin samples excised from cadavers demonstrated TEWL and skin conductance to be poorly correlated with one another,^10^ which yields indirect evidence that supports this distinction of barriers. Given the reciprocal relationship between skin conductance and resistance, this study is relevant by way of ionic influences on skin resistance. Factors that influence skin resistance include surface electrolyte concentrations due to their ability to conduct electricity and therefore lower resistance, as well as skin moisture levels, contact pressure, contact area, and the presence of cuts/pinprick wounds.^11^ Were the water barrier and electrolyte barrier synonymous, a positive correlation would have been seen between TEWL and skin conductance values.

The epidermal water barrier has been extensively studied when compared to the electrolyte barrier. The water barrier site has long been established to exist within the stratum corneum.^12^, ^13^ Subsequent investigation revealed that lipid lamellae comprised mostly of ceramides, cholesterol, and free fatty acids fill the intercellular spaces between corneocytes of the stratum corneum,^14^, ^15^ and function to retard water loss.^16^, ^17^ Less attention to detail has been given to elucidating the site and constituents of the electrolyte barrier, although a set of more recent experiments performed by Kirschner et al.^18^ on human keratinocyte cell cultures has emphasized the role of tight junctions in resisting Na^+^, Cl^–^, and Ca^2+^ ion permeability.

Cultured human keratinocytes were bathed in a high‐calcium solution of 1.8 mM to induce expression of tight junctions. Ussing chambers corrected for the bathing solution were then used to measure transcellular resistance, paracellular resistance, and the combined resistance of the two, the transepithelial resistance, as well as the permeability of the aforementioned ions across the cultured monolayer. Individual knockdown models of tight junction proteins claudin‐1, claudin‐4, occludin, and zonula occludens were also tested against the same parameters.

At 48 h in solution, transepithelial resistance of cultured keratinocytes was significantly increased compared to the starting point, as were paracellular and transcellular resistance. Paracellular resistance, which reflects movement through the paracellular pathway in which tight junctions regulate, was largely responsible for the significant increase in transepithelial resistance; transcellular resistance increased only slightly. Additionally, the permeability of Na^+^, Cl^–^, and Ca^2+^ ions all decreased significantly. In knockdown models, transepithelial resistance, paracellular resistance, and transcellular resistance significantly decreased for all models except in claudin‐4. Claudin‐4 knockdown significantly decreased transepithelial and paracellular resistance without altering transcellular resistance. The permeability of Na^+^, Cl^–^, and Ca^2+^ ions was significantly increased in all knockdown models. Altogether, the experiments here provide evidence that tight junctions may comprise the electrolyte barrier in skin, seeing as their formation reduced ion permeability, and knockdown of their component proteins enhanced ion permeability. Further, these findings would imply that the site of the electrolyte barrier resides in the stratum granulosum, given that tight junctions in the epidermis largely exist between these granular cells.^19^

Regardless of where or what the electrolyte barrier may be, the reviewed works evaluating transepidermal ion flux in response to barrier perturbation suggest that the percutaneous egression of ions may serve as a valuable marker for determining skin barrier integrity. Not only is there continued potential for this technique to be utilized in a laboratory with ion‐specific electrodes, but Ichimura et al.^20^ provide proof of concept for the development of a portable biosensor that would enable a more widespread application of transepidermal ion flux to take place outside the traditional research setting. The investigators reported the development of a novel, screen‐printed chloride ion sensor utilizing a hydrogel touchpad sensitive enough to quantify chloride ions diffusing out of the skin via direct contact at rest, whereas traditional wearable sensors require active perspiration induced by exercise, temperature control, or cholinergic stimulation to obtain readings. The novel sensor is comprised of various layers, but chiefly important is the inclusion of a reference electrode ionically connected to the chloride‐specific sensing electrode by an electrolyte layer of baked KCl aqueous solution. On top of the sensor is a 4% by weight agarose gel in phosphate buffer solution that serves as a touchpad and is surrounded by a silicon rubber sheet. Finger placement onto the hydrogel touchpad allows for extraction of chloride ions from the epidermis that subsequently interacts with the electrodes via the hydrogel, resulting in a detectable change in the potential using a voltmeter. This change in potential can then be used to calculate chloride concentrations. With the advent of this technology allowing for the passive and inexpensive detection of chloride ions outside the laboratory setting, the research‐ and clinical‐based applications of quantifying ion flux through the skin are expected to grow, particularly as these sensors are further refined to improve accuracy.

While the experimental studies included in this review evaluated the outward flux of multiple ions, including calcium, potassium, sodium, and hydrogen, it is difficult to draw comparisons of their unique utilities for assessing barrier function due to a lack of data. Additionally, exact mechanisms of ion egression beyond passive diffusion are also unclear and require further investigation to understand their contribution to transepidermal ion flux. Advancements in these areas would likely broaden the clinical applications of quantifying ion flux.

Beyond the percutaneous egression of ions, Fortenbach^21^ examined a drug's physicochemical properties in relation to its delivery to the skin using cantharidin blister fluid. There was no statistically significant correlation between a drug's partition coefficient and the fraction penetrating into the epidermal interstitial fluid, which was calculated by dividing AUC_blister_ by AUC_serum_. There was, however, a strong linear correlation between total drug concentration in serum and interstitial fluid. Fortenbach et al. described the percutaneous egression of some drugs but did not detail where the drug barriers occur.

The deuterated water provides another avenue of understanding barrier formation and possible perturbation. Emson et al.^22^ evaluated keratin synthesis in human skin of psoriatic subjects who were orally dosed 50 ml^2^ H_2_O twice daily for 16–38 days. Tape‐strippings were collected every 2–5 days, and protein content was assessed. Deuterium‐labeled keratin appeared within 3–8 days of administration in lesional skin versus 10–20 days in unaffected skin, demonstrating rapid replacement of stratum corneum in lesional skin and the role of deuterated water in detecting barrier formation.

A final skin barrier to consider is the barrier to outward diffusion of oxygen and carbon dioxide, which has previously been measured by Cunico et al.^23^ and Wilson et al.^24^

## 5 CONCLUSION

Skin has multiple barrier functions. TEWL is widely used to measure skin barrier function, but it is not the only method available to do so. Literature quantifying the percutaneous egression of ions in response to barrier disruption, discussed in this overview, has demonstrated that this technique may be a feasible alternative for assessing barrier function. Continued efforts should be made to progress transepidermal ion flux as an affordable, uncomplicated, and non‐invasive method of barrier assessment. Additionally, there is evidence demonstrating that a damaged water barrier does not necessitate a damaged electrolyte barrier. This is potentially due to varying locations and constituents for each distinctive barrier. Where and what the electrolyte barrier may be lies an area open to vast exploration, although recent work points to stratum granulosum tight junctions as a primary constituent. We suspect that much remains to be learned regarding other barriers and mechanisms. The ion data, here, alongside work by Fortenbach et al.,^21^ Cunico et al.,^23^ and Wilson et al.,^24^ provide impetus to enlarge our barrier understandings. We suspect that the situation of skin barriers may resemble that of our knowledge of transporters; the first transporter superfamily, the ATP‐binding cassette transporters, was recognized 24 years ago,^25^ and today over 400 transporters have been discovered.^26^

## CONFLICT OF INTEREST

The authors declare that there is no conflict of interest that could be perceived as prejudicing the impartiality of the research reported.
